# Gastrodin Alleviates Angiotensin II-Induced Hypertension and Myocardial Apoptosis via Inhibition of the PRDX2/p53 Pathway In Vivo and In Vitro

**DOI:** 10.3390/ph17091200

**Published:** 2024-09-12

**Authors:** Nanhui Xu, Qiurong Xie, Youqin Chen, Jiapeng Li, Xiuli Zhang, Huifang Zheng, Ying Cheng, Meizhu Wu, Aling Shen, Lihui Wei, Mengying Yao, Yanyan Yang, Thomas J. Sferra, Anjum Jafri, Yi Fang, Jun Peng

**Affiliations:** 1 Academy of Integrative Medicine, Fujian University of Traditional Chinese Medicine, Fuzhou 350122, China; 1191010006@fjtcm.edu.cn (N.X.); 2020025@fjtcm.edu.cn (Q.X.); 1201100007@fjtcm.edu.cn (X.Z.); c2020020@fjtcm.edu.cn (H.Z.); 2181133010@fjtcm.edu.cn (Y.C.); 1211006008@fjtcm.edu.cn (M.W.); 2018025@fjtcm.edu.cn (A.S.); weilihui@fjtcm.edu.cn (L.W.); 2201100021@fjtcm.edu.cn (M.Y.); 2011016@fjtcm.edu.cn (Y.Y.); 2 Fujian Key Laboratory of Integrative Medicine on Geriatrics, Fujian University of Traditional Chinese Medicine, Fuzhou 350122, China; 3 Fujian Collaborative Innovation Center for Integrative Medicine in Prevention and Treatment of Major Chronic Cardiovascular Diseases, Fuzhou 350122, China; 4 Innovation and Transformation Center, Fujian University of Traditional Chinese Medicine, Fuzhou 350122, China; 5 Department of Pediatrics, Rainbow Babies and Children’s Hospital, Case Western Reserve University School of Medicine, Cleveland, OH 44106, USA; yxc571@case.edu (Y.C.); thomas.sferra@uhhospitals.org (T.J.S.); axj21@case.edu (A.J.); 6 Department of Physical Education, Fujian University of Traditional Chinese Medicine, Fuzhou 350122, China; 2009050@fjtcm.edu.cn

**Keywords:** gastrodin, hypertension, cardiac protection, myocardial apoptosis, angiotensin II, PRDX2/p53 pathway

## Abstract

Gastrodin, a highly potent compound found in the traditional Chinese medicine *Gastrodia elata* Blume, exhibits significant antihypertensive properties. However, its role and the mechanism behind its protective effects on hypertensive cardiac conditions are not well understood. This study aims to investigate the cardiac protective effects and underlying mechanisms of gastrodin in angiotensin II (Ang II)-induced hypertensive models, both in vivo and in vitro. Treatment with gastrodin significantly decreased blood pressure and the heart weight/tibial length (HW/TL) ratio and attenuated cardiac dysfunction and pathological damage in Ang II-infused C57BL/6 mice. RNA sequencing analysis (RNA-seq) revealed 697 up-regulated and 714 down-regulated transcripts, along with 1105 signaling pathways, in Ang II-infused C57BL/6 mice following gastrodin treatment, compared to Ang II-induced hypertensive mice. Furthermore, the analyses of the top 30 Gene Ontology (GO) terms and Kyoto Encyclopedia of Genes and Genomes (KEGG) pathway indicated significant enrichment in apoptosis and the peroxiredoxin 2 (PRDX2)/p53 pathway. Consistently, gastrodin treatment significantly reduced myocardial apoptosis in both the cardiac tissues of Ang II-induced hypertensive mice and Ang II-stimulated H9c2 cells. Additionally, gastrodin treatment significantly decreased the protein levels of PRDX2, p53, cleaved caspase-3, cleaved caspase-9, and Bax/Bcl-2 ratio in the cardiac tissues of Ang II-infused mice and H9c2 cells stimulated with Ang II. In conclusion, gastrodin treatment can mitigate hypertension-induced myocardial apoptosis in hypertensive mice by inhibiting the PRDX2/p53 pathway.

## 1. Introduction

Hypertension is a primary cardiovascular epidemic in both developed and developing countries, which is always accompanied by a variety of complications in the course of the disease [1]. Hypertension-induced cardiac injury is recognized as a leading cause of organ damage and mortality worldwide [2]. Studies have reported that cardiac injury includes cardiac hypertrophy, which is accompanied by cellular proliferation and hypertrophy, myocardial apoptosis, and inflammation [3,4]. 

p53 plays a crucial role in the endogenous apoptosis pathway, functioning as a transcription factor to activate many downstream apoptotic genes [5]. Continuous accumulation of p53 during cardiac hypertrophy in endothelial cells and myocardial tissues results in impaired angiogenesis and myocardial injury, which contributes to the progression from hypertrophy to heart failure [6,7]. Peroxiredoxin 2 (PRDX2), a member of the peroxiredoxin family, is associated with the p53-dependent apoptotic pathway in human hepatoma cells [8]. Therefore, we hypothesize that the PRDX2/p53-dependent apoptotic pathway may be involved in hypertensive myocardial injury. 

A Chinese traditional medicine, *Gastrodia elata* Blume (commonly known as “Tian Ma” in Chinese), exhibits significant therapeutic efficacy in reducing blood pressure levels [9]. Gastrodin, a major component of *Gastrodia elata* Blume, is a phenolic glycoside [10] (Figure S1) with excellent oral bioavailability, exceeding 80% [11]. Remarkably, gastrodin is not rapidly eliminated from the body; it remains detectable in rat plasma for up to 8 h after oral administration [12]. Gastrodin has been used in the treatment of hypertension, Parkinson’s disease and other conditions [13,14,15,16]. Moreover, it can improve cardiac hypertrophy in mice with transverse aortic constriction and reduce cardiomyocyte hypertrophy induced by both transverse aortic constriction and phenylephrine [17,18]. Gastrodin also inhibited cell apoptosis induced by homocysteine, interleukin-1 beta (IL-1β), high glucose, and oxidative stress through various targets [19,20,21,22]. However, the protective effect of gastrodin on hypertensive myocardial apoptosis and its underlying mechanism has yet to be clarified. By using echocardiography, RNA sequencing (RNA-seq), and various other technologies, our study investigates whether gastrodin can effectively protect the heart from cardiac injury and elucidate its underlying mechanisms.

## 2. Results

### 2.1. Gastrodin Reduces the Blood Pressure in Ang II-Induced Hypertensive Mice

**Figure 1:**
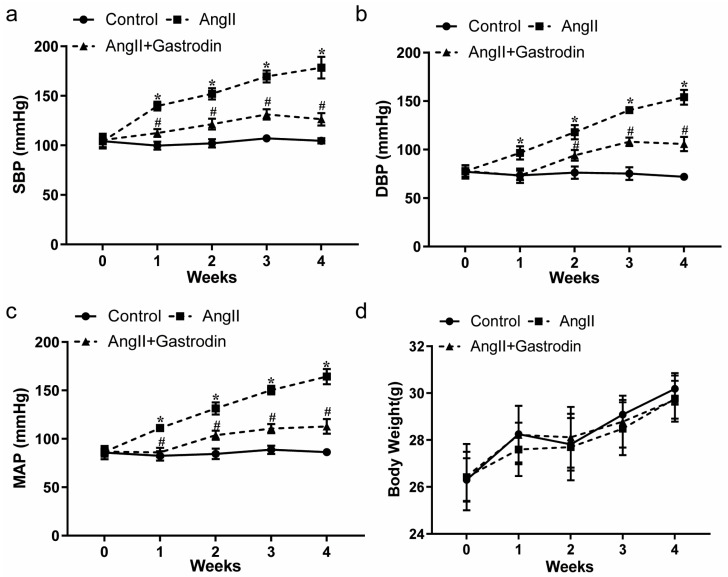
The effects of gastrodin on blood pressure in Ang II-infused mice. Blood pressure was measured in mice from each group using the tail-cuff plethysmograph method once a week for a total of 4 weeks. (**a**) Systolic blood pressure (SBP), (**b**) diastolic blood pressure (DBP), (**c**) mean arterial pressure (MAP). (**d**) Body weight of mice from each group. All values are presented as mean ± SD, *n* = 6. * *p* < 0.05 vs. the Control group; # *p* < 0.05 vs. the Ang II group.

With the general condition of the mice—including mental state, diet, drinking, defecation, and urination—showing no abnormalities across all groups, blood pressure measurements revealed a significant increase in systolic blood pressure (SBP) (Figure 1a, Table 1), diastolic blood pressure (DBP) (Figure 1b, Table 2), and mean arterial pressure (MAP) (Figure 1c, Table 3) in Ang II-induced hypertensive mice. This increase was significantly mitigated by gastrodin treatment (Figure 1a–c). Additionally, there were no significant differences in weight changes among the groups (Figure 1d). 

These findings further revealed that gastrodin exerts a consistent and effective antihypertensive effect in angiotensin II (Ang II)-induced hypertensive mice. 

### 2.2. Gastrodin Attenuates Cardiac Dysfunction and Pathological Damage in Ang II-Induced Hypertensive Mice

**Figure 2:**
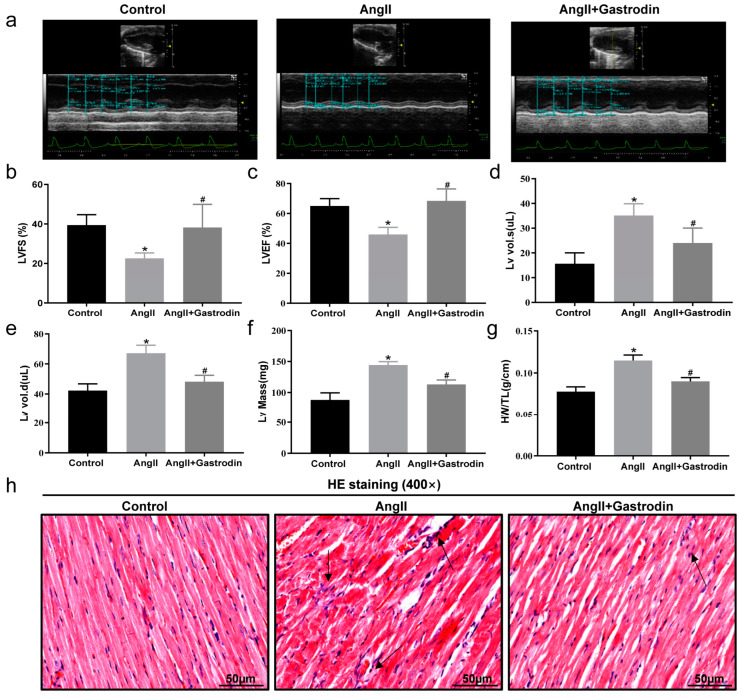
Gastrodin alleviates Ang II-induced cardiac dysfunction. (**a**) Representative images of M-mode echocardiography from each group of mice. (**b**) LVFS, (**c**) LVEF, (**d**) LV vol.s, (**e**) LV vol.d, (**f**) LV Mass. (**g**) The ratio of HW/T in each group. (**h**) Cardiac tissues of mice from each group were stained with HE, and representative images were taken at a magnification of ×400. The Arrow indicates infiltrating inflammatory cells in cardiac tissue. All values are presented as mean ± SD, *n* = 6. * *p* < 0.05 vs. the Control group; # *p* < 0.05 vs. the Ang II group.

We further evaluated cardiac function using cardiac ultrasound analysis (Figure 2a). In Ang II-infused mice, left ventricle fractional shortening (LVFS) (Figure 2b) and left ventricle ejection fraction (LVEF) (Figure 2c) were significantly decreased, while left ventricle volume systole (LV Vol. s) (Figure 2d), left ventricle volume diastole (LV Vol. d) (Figure 2e), and corrected left ventricular mass (LV Mass) were increased (Figure 2f). These changes were all attenuated following gastrodin treatment (Figure 2b–f). These results demonstrated the cardioprotective effect of gastrodin on Ang II-induced hypertensive mice. Further comparison of the heart weight/tibial length (HW/TL) ratio among the groups showed a significant increase after Ang II infusion, which was alleviated by gastrodin treatment (Figure 2g). Consistently, hematoxylin and eosin (H&E) staining revealed that gastrodin treatment attenuated the pathological changes in the cardiac tissues of Ang II-induced hypertensive mice, including disorganization of myocardium tissues, nuclear deformation, pyknosis, and infiltration of inflammatory cells (Figure 2h). These results indicated that gastrodin treatment significantly attenuates cardiac dysfunction and pathological changes in Ang II-infused mice.

### 2.3. The Differentially Expressed Transcripts (DETs) and Pathway Enrichment in the Cardiac Tissues of Ang II-Induced Hypertensive Mice Following Gastrodin Treatment

**Figure 3:**
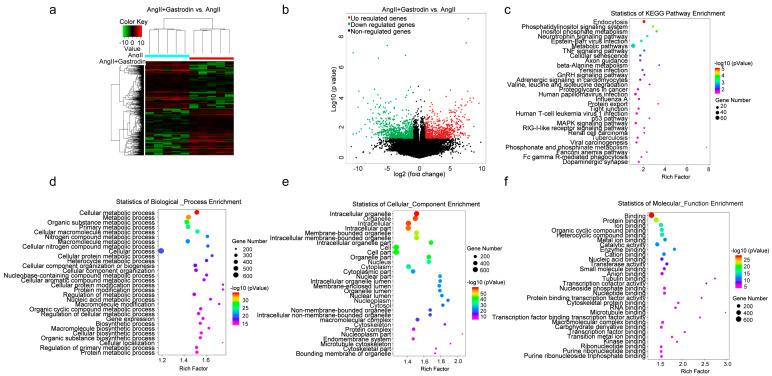
RNA-seq analysis of differentially expressed genes and signaling pathways in the cardiac tissues of Ang II-infused mice after gastrodin treatment. (**a**) Hierarchical clustering plots and (**b**) volcano plots were used to compare gene expression profiles (|fold change| ≥ 2, *p* < 0.05). (**c**) The overlapping KEGG pathways in the comparisons of Ang II + gastrodin vs. Ang II. The top 30 enriched items of the (**d**) biological processes, (**e**) cellular composition, and (**f**) molecular function. GO analysis was performed based on the DETs from comparisons of Ang II + gastrodin vs. Ang II. All values are represented as mean ± SD, *n* = 6.

To further investigate the molecular mechanism by which gastrodin alleviates hypertension-induced cardiac injury, RNA-seq was performed to detect transcriptional alterations in the Ang II and Ang II + gastrodin groups. The cluster maps (Figure 3a) and volcano maps (Figure 3b) created based on differentially expressed transcript (DET) levels (NCBI GEO: GSE193504) revealed 697 up-regulated and 714 down-regulated transcripts (fold-change ≥ 2, *p* < 0.05) after gastrodin treatment, compared to the Ang II group. 

Furthermore, 1105 signaling pathways were enriched in the Ang II+ gastrodin group, compared to the Ang II group. The top 30 Kyoto Encyclopedia of Genes and Genomes (KEGG) pathways enriched based on the DETs between Ang II + gastrodin and Ang II groups indicated that endocytosis, the phosphatidylinositol signaling system, the phosphatidylinositol signaling system, the neurotrophin signaling pathway, metabolic pathways, the TNF signaling pathway, and adrenergic signaling in cardiomyocytes were enriched by gastrodin treatment (Figure 3c). Additionally, the p53 pathway, strongly associated with myocardial apoptosis, was also identified among the top 30 KEGG pathways, consistent with our hypothesis.

To delve deeper into the mechanism of gastrodin treatment, Gene Ontology (GO) analysis was conducted to identify different biological functions in terms of biological processes (BPs), cellular composition (CC), and molecular function (MF) based on the DETs. Several metabolic processes were enriched in the BP category (Figure 3d). CC analysis revealed that DETs were concentrated in intracellular, cell part, and intracellular organelle (Figure 3e). Protein binding, ion binding, and organic cyclic compound binding were enriched in the MF category (Figure 3f). Given that 17 out of the 30 biological processes were associated with metabolic processes (Figure 3c), the metabolic pathways identified in the KEGG pathway enrichment analysis were further examined. Interestingly, PRDX2, which was significantly down-regulated in the cardiac tissues of the Ang II + gastrodin group, was identified in the “TP53 regulates metabolic genes” pathway, a subset of the metabolic pathways in the KEGG pathway enrichment analysis. While studies have indicated that PRDX2 regulates the p53 pathway in the proliferation of either cancer cells or trophoblast cells during recurrent miscarriage, its role in hypertensive cardiac apoptosis remains unknown [8,23,24,25]. Thus, these findings suggested a need to investigate how gastrodin regulates cardiac apoptosis through the PRDX2/p53 pathway.

### 2.4. Gastrodin Reduces Ang II-Induced Myocardial Apoptosis In Vivo 

**Figure 4:**
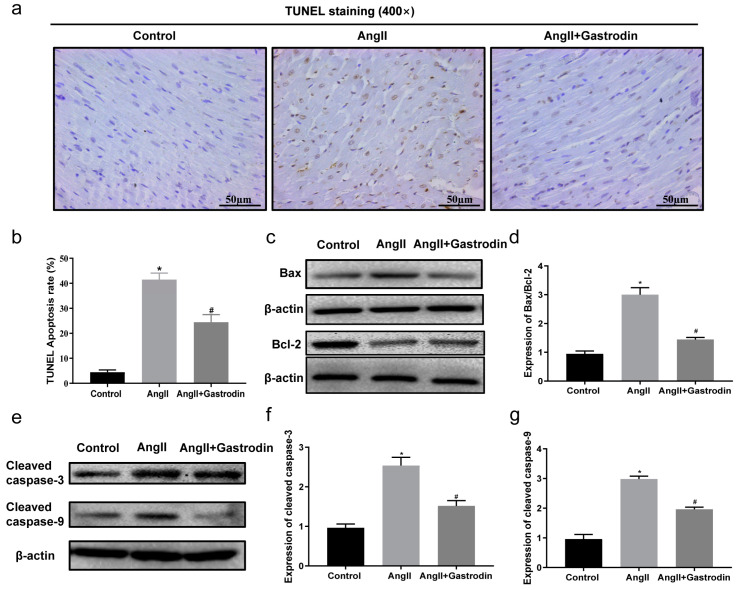
Gastrodin reduces Ang II-induced cardiac apoptosis in the cardiac tissues of Ang II-infused mice. (**a**) Cardiac tissues of mice from each group were stained with TUNEL (*n* = 6), and representative images were taken at a magnification of ×400. (**b**) The percentage of TUNEL-positive cells in each group was analyzed. (**c**) Western blot analysis was performed to determine the protein expression of Bax and Bcl-2. (**d**) The ratio of Bax/Bcl-2 was analyzed by Image J (version 2022). (**e**) Western blot analysis was performed to determine the protein expression of cleaved caspase-3 and cleaved caspase-9. (**f**,**g**) The ratio of cleaved caspase-3 and cleaved caspase-9 were analyzed by Image J (version 2022) and β-actin was used as the internal Control. All values are represented as mean ± SD. * *p* < 0.05 vs. the Control group; ^#^ *p* < 0.05 vs. the Ang II group.

To validate the results from the KEGG pathway analysis, terminal deoxynucleotidyl transferase-mediated nick end labeling (TUNEL) staining was used to assess myocardial apoptosis in the cardiac tissues. The results showed a significant increase in the percentage of TUNEL-positive stained cells in the cardiac tissues of Ang II-infused mice, which was reduced after gastrodin treatment (Figure 4a,b). Furthermore, Western blotting revealed that the protein ratio of Bax/Bcl-2 (Figure 4c,d) and levels of cleaved caspase-3 and cleaved caspase-9 (Figure 4e–g) were up-regulated by Ang II infusion, all of which were reversed by gastrodin treatment. These findings indicated that gastrodin attenuated myocardial apoptosis in Ang II-induced hypertensive mice.

### 2.5. Gastrodin Attenuates Ang II-Induced Apoptosis in H9c2 Cells

**Figure 5:**
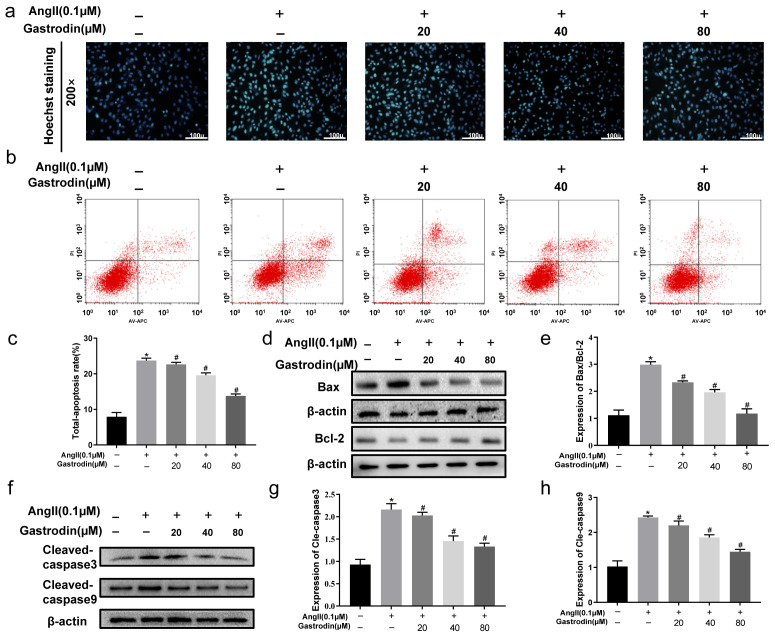
Gastrodin reduces Ang II-induced cardiac apoptosis in Ang II-stimulated H9c2 cells. (**a**) H9c2 cells from each group were stained with Hoechst. Representative images were taken at a magnification of ×200. (**b**) Representative dot plot of Annexin V and PI-stained cells. The result involved setting up quadrant gates based on negative cell groups, as well as using single staining with Annexin V and PI. Specifically, Annexin V (−)/PI (−) represents normal living cells, Annexin V (+)/PI (−) represents apoptotic cells at early stage, Annexin V (−)/PI (+) represents apoptotic cells at late stage, and Annexin V (+)/PI (+) represents cells undergoing necrosis or with severe mechanical damage. (**c**) The percentage of total apoptosis in H9c2 cells. (**d**) Western blot analysis was performed to determine the protein expression of Bax and Bcl-2. (**e**) The ratio of Bax/Bcl-2 was analyzed by Image J (version 2022). (**f**) Western blot analysis was performed to determine the protein expression of cle-caspase-3 and cle-caspase-9. (**g**,**h**) The ratio of cle-caspase-3 and cle-caspase-9 were analyzed by Image J (version 2022), and β-actin was used as the internal Control. All values are represented as mean ± SD, *n* = 3. * *p* < 0.05 vs. the Control group; ^#^ *p* < 0.05 vs. the Ang II group.

The Hoechst assay was next performed to evaluate the effect of gastrodin on apoptosis in Ang II-stimulated H9c2 cells. The results showed that Ang II stimulation significantly increased the percentage of apoptosis cells compared to Control cells, which was reduced with gastrodin treatment (Figure 5a). Consistently, an Annexin V/propidium iodide (PI) assay confirmed that Ang II stimulation led to a significant increase in apoptotic cells, which decreased following treatment with different concentrations of gastrodin (Figure 5b,c). Additionally, the protein ratio of Bax/Bcl-2 (Figure 5d,e) and the levels of cleaved caspase-3 (Figure 5f,g) and cleaved caspase-9 (Figure 5f,h) were significantly elevated with Ang II stimulation, but these levels were also markedly reduced by gastrodin treatment. These results demonstrated that gastrodin attenuates Ang II-induced apoptosis in vitro. In summary, these results suggested that gastrodin could reduce hypertension-induced myocardial apoptosis in hypertensive mice induced by Ang II in vivo, as well as H9c2 cells stimulated by Ang II in vitro.

### 2.6. Gastrodin Inhibits PRDX2/p53 Pathway Activation in Ang II-Induced Hypertension In Vivo and In Vitro

**Figure 6:**
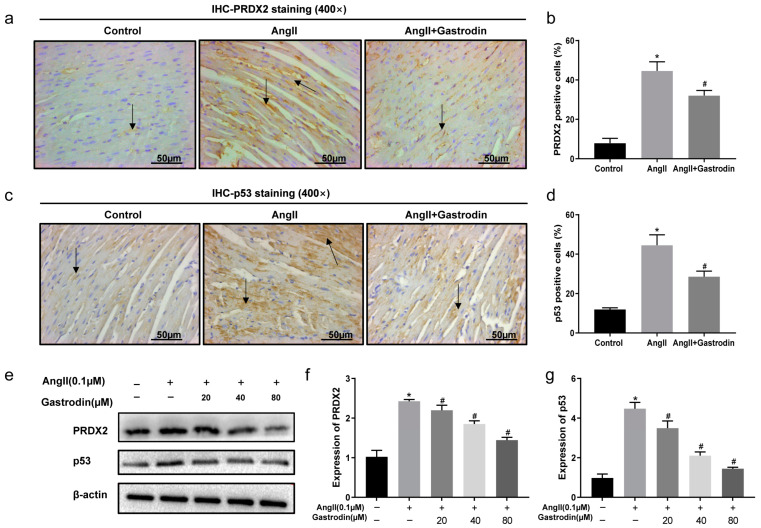
Gastrodin inhibits Ang II-induced PRDX2/p53 pathway activation in vivo and in vitro. Representative images of PRDX2 (**a**) and p53 (**c**) protein stained by IHC analysis (*n* = 6). Representative images were taken at a magnification of ×400. Arrow indicates PRDX2 protein expressed in cardiac tissue. PRDX2 (**b**) and p53 (**d**) protein expression in the cardiac tissues of mice from each group was analyzed. (**e**–**g**) Western blot analysis was performed to determine the protein expression of PRDX2 and p53 in H9c2 cells from each group. All values are represented as mean ± SD. * *p* < 0.05 vs. the Control group; ^#^ *p* < 0.05 vs. the Ang II group.

Based on the enrichment from the KEGG pathway analysis, the alterations in PRDX2 and p53 protein levels were subsequently evaluated both in vivo and in vitro following Ang II stimulation and gastrodin treatment. The results revealed that Ang II stimulation induced a significant increase in the expression of PRDX2 and p53, which were attenuated by gastrodin treatment both in vivo and in vitro (Figure 6a,b,e,f). Similarly, gastrodin reduced the elevated levels of PRDX2 and p53 protein induced by Ang II stimulation in both models (Figure 6c–e,g). These findings implied that gastrodin treatment attenuates Ang II-induced PRDX2/p53 pathway activation.

## 3. Discussion

Hypertensive cardiac injury poses a severe threat to patients’ lives [26]. Therefore, identifying effective methods to reduce blood pressure and minimize cardiac damage is a critical priority. Clinical studies have revealed that gastrodin, a component of *Gastrodia elata* Blume, when used as an adjunct to conventional hypertension treatments—including ACEIs (Angiotensin-Converting Enzyme Inhibitors), CCBs (Calcium Channel Blockers), and ARBs (Angiotensin Receptor Blockers)—effectively reduce blood pressure [9,27,28,29]. It has been proven to have a high safety profile and potential for long-term administration at larger doses in acute and subacute toxicity experiments [30]; however, its use in high doses or in patients over 45 years old may lead to adverse drug reactions, primarily gastrointestinal discomfort and skin allergies. Thus, effective monitoring through the clinical standardized application and close observation of patients within the first two hours after medication administration can significantly enhance safety [31].

Previous studies have also revealed the antihypertensive effect of gastrodin in various animal models [32,33,34,35]. Our previous research using female spontaneous hypertensive rats and angiotensin II-induced male mice explored the underlying mechanism of gastrodin [33,34]. We found that gastrodin lowered blood pressure, including SBP, DBP, and MAP in Ang II-infused mice. Additionally, it partially restored hypertensive cardiac dysfunction by increasing left ventricular ejection fraction and left ventricular fractional shortening, decreasing left ventricular diastolic and systolic volumes, and correcting left ventricular mass. However, further research is needed to evaluate the antihypertensive effects of gastrodin in female mice, as well as in other genetic backgrounds or environmental conditions (such as diet, stress, etc.), to supplement our findings.

Previous studies have reported that gastrodin effectively alleviates hypertensive cardiac hypertrophy and fibrosis by regulating mitochondrial permeability and inhibiting the production of reactive oxygen species (ROS) [30,36,37]. Gastrodin has been shown to reduce the levels of oxidative stress biomarkers such as malondialdehyde (MDA), lactate dehydrogenase (LDH), and reactive oxygen species (ROS), thereby protecting human umbilical vein endothelial cells (HUVECs) from homocysteine-induced injury [19]. Additionally, gastrodin down-regulated the expression of NOX-2, a subunit of the ROS-producing enzyme NADPH oxidase involved in oxidative reactions [38,39]. Our study revealed that gastrodin alleviated cardiac pathological changes such as cardiac fiber disorganization and enlarged interfibrillar gaps. These findings indicated that gastrodin can reduce blood pressure and cardiac damage in hypertension. However, these findings were observed only in mice; extensive clinical trials are necessary to confirm whether gastrodin can also reduce heart damage in hypertensive patients. Furthermore, the MAPK pathway, which can be activated by ROS [40], was enriched in the differential KEGG pathway analysis between the Ang II + gastrodin and Ang II groups (Figure 3c). Based on this evidence, we hypothesize that gastrodin may exert an antioxidative stress effect in angiotensin II-induced hypertension, which could be further validated in future research. Cardiac injury resulting from hypertension not only includes cardiac hypertrophy and fibrosis but also inflammation and myocardial apoptosis [41,42,43,44,45]. Previous studies have shown that gastrodin alleviates inflammatory injury by suppressing NLRP3 in septic shock mice cardiomyocytes [46]. Other studies have reported that gastrodin exerts an anti-inflammatory effect by inhibiting Ang II production in the renin-angiotensin system (RAS) [47,48,49,50]. For instance, gastrodin has been shown to suppress proinflammatory mediators such as TNF-α, iNOS, and IL-1β, which are activated by Ang II [39,51]. Additionally, IGF2R, known to activate anti-inflammatory reactions, was down-regulated by gastrodin in its cardioprotective action [52,53]. Our study demonstrated that gastrodin attenuates cardiac dysfunction and pathological changes, including the infiltration of inflammatory cells in Ang II-infused mice. The enrichment of the TNF-α signaling pathway following gastrodin treatment suggested that anti-inflammatory effects may contribute to gastrodin’s protective role in angiotensin II-induced hypertension (Figure 3c). Further research is necessary to determine the anti-inflammatory effect of gastrodin in Ang II-induced hypertensive mice by assessing serum inflammation marker levels or the activation of the TNF-α signaling pathway. We believe that such findings would help us to better understand the protection of gastrodin in Ang II-induced hypertension.

Gastrodin also prevents oxidative stress-induced apoptosis by inhibiting mPTP (mitochondrial permeability transition pore) opening and the NFE2/HOX1 pathway [54,55]. Furthermore, gastrodin has been shown to protect myocardial cell apoptosis from ischemia–reperfusion injury by regulating Bax and Bcl-2 [15]. It inhibited apoptosis in response to homocysteine, IL-1β, and high glucose by modulating the PI3K/AKT or NF-κB pathway [19,20,21]. A loss of p53 has been found to counteract myocardial ischemia/reperfusion injury, while its activation accelerates the deterioration of left ventricular function [56]. Overexpression of p53 during myocardial apoptosis is often accompanied by an increased Bax/Bcl-2 protein ratio and elevated levels of cleaved caspase-9 and cleaved caspase-3 [57]. Therefore, inhibiting the activation of these apoptosis-related proteins (p53, Bax/Bcl-2 protein ratio, and caspase-3/9) is a common strategy in several anti-myocardial apoptotic therapies [58,59]. Gastrodin has been reported to inhibit p53 activation in apoptosis induced by LPS/D-GalN (Lipopolysaccharide/D-galactosamine) or glutamate [60,61]. Collectively, these findings indicate that p53 activation, along with up-regulation of the Bax/Bcl-2 ratio, is closely related to myocardial apoptosis. Our current study provides additional evidence that Ang II stimulation led to a notable increase in the apoptosis rate of myocardial cells, the Bax/Bcl-2 protein ratio, and the protein levels of p53, cleaved caspase-9 and cleaved caspase-3 in cardiac tissues and H9c2 cells. Gastrodin intervention mitigated these effects, demonstrating that gastrodin alleviates myocardial apoptosis by regulating the p53 pathway. 

Moreover, the DETs and pathways obtained from RNA-seq data provided insights into gastrodin’s protective mechanisms in cardiac injury. Various metabolic processes identified through GO analysis suggested that gastrodin reduces hypertension by improving metabolic processes, which are often disrupted in hypertensive individuals [62]. Interestingly, PRDX2, a member of the peroxiredoxin family involved in metabolic processes, was one of the DETs identified in our study. Targeting PRDX2 is a strategy to combat oxidative stress, excessive ROS release, and apoptosis [63]. In an acute myocardial infarction model, PRDX2 was shown to enhance cardiomyocyte inflammation and myocardial hypertrophy [64]. Additionally, PRDX2 protein levels were up-regulated in human carotid artery tissues with atherosclerosis compared to normal carotid artery tissues, suggesting that PRDX2 up-regulation may be a stress response [65]. Our results showed myocardial hypertrophy and cardiomyocyte apoptosis in Ang II-induced cardiac tissue of mice, along with an upward trend in PRDX2 protein expression. This supports the evidence that PRDX2 is involved in the regulation of myocardial hypertrophy and cardiomyocyte apoptosis in hypertension [64,65]. Our findings suggest that suppressing Ang II-induced activation of the PRDX2 and p53 signaling pathway may be a potential mechanism by which gastrodin attenuates apoptosis in hypertensive cardiac injury. However, whether gastrodin specifically regulates the PRDX2/p53 pathway, or if its effects are part of a broader antioxidant or anti-apoptotic response, remains to be fully elucidated. To address this, further research is needed to conduct in-depth experiments using the activator or inhibitor PRDX2/p53 pathway to observe the specific underlying mechanisms and to better understand the regulatory role of gastrodin on the PRDX2/p53 pathway.

Additionally, among 1105 differential signaling pathways significantly enriched between the Ang II group and the Ang II + gastrodin group, several viral infection-related pathways, such as the RIG-I-like receptor signaling pathway, viral carcinogenesis, as well as Influenza A, Epstein–Barr virus infection, Human T-cell leukemia virus 1 infection and Human papillomavirus infection. These findings were consistent with studies on the antiviral effects of gastrodin [66,67]. Moreover, gastrodin has been reported to improve immune responses mediated by CD8+ T cells, activate CD4+ T cells, and reprogram macrophages [68,69,70]. In our study, we also found that gastrodin regulates Fc gamma R-mediated phagocytosis, which is known to mediate the phagocytic activity of the cytoplasmic membrane in the mononuclear phagocytic system and other biological reactions. Additionally, modern pharmaceutical studies have shown that gastrodin can regulate blood pressure by inhibiting the renin–angiotensin–aldosterone system (RAAS) system and preventing the influx and release of Ca^2+^. Specifically, gastrodin can inhibit sympathetic nervous system stimulation and reduce the concentrations of serum angiotensin II, aldosterone (ALD), and AT1R, effectively inhibiting RAAS and PPARγ to reduce blood pressure [35,71]. In our study, the neurotrophin signaling pathway, which was enriched in the differential KEGG pathway analysis between Ang II + gastrodin and Ang II, is consistent with previous studies. These results further suggest that gastrodin regulates the nervous system [72,73,74]. Furthermore, many unreported differential pathways may be closely related to the antihypertensive mechanism of gastrodin. However, what is the exact mechanism for each differential pathway? Further in-depth exploration is needed in future research to fully understand these mechanisms.

## 4. Materials and Methods

### 4.1. Reagents and Consumables

High-glucose Dulbecco’s modified Eagle’s medium (DMEM) (cat. no. c11995500bt), penicillin–streptomycin (cat. no. sv30010), fetal bovine serum (FBS, cat. no.10099141c), bicinchoninic acid (BCA) protein assay kit (cat. no.23228), and trypsin–EDTA (cat. no. 25200072) were sourced from Thermo-Fisher Scientific (Waltham, MA, USA). The osmotic mini-pump (cat. no. Alzet 2004) was purchased from DURECT Corporation (Mountain View, CA, USA). Cell lysis buffer for Western and IP were obtained from Beyotime Biotechnology (cat. no P0013, Shanghai, China). Terminal deoxynucleotidyl transferase-mediated nick end labeling (TUNEL) apoptosis detection kit (cat. no. MK1025) was obtained from Boster Biological Technology Co, Ltd. (Wuhan, China). Annexin V-AbFlour^TM^ 647 Apoptosis Detection Kit (cat. no. KTA0004) was obtained from Abbkine Scientific Co., Ltd. (Wuhan, China). Ang II (cat. no. ab120183) was purchased from Abcam (Cambridge, UK). Antibody against PRDX2 (cat. no. 49273-1) was obtained from Signalway Antibody (Nanjing, China). Antibodies against Bcl-2 (cat. no. 12789-1-AP) and p53 (cat. no. 10442-1-AP) were obtained from Proteintech (Rosemont, IL, USA). Antibodies for cleaved caspase-3 (cat. no. 9662), cleaved caspase-9 (cat. no. 9508), Bax (cat. no. 2772), β-actin (cat. no. 3700), mouse IgG (cat. no. 7076), and rabbit IgG (cat. no. 7074) were acquired from Cell Signaling Technology (Danvers, MA, USA). Gastrodin (purity ≥98%, cat. no. 62499-27-8) was purchased from Yuanye bio-technology Co., Ltd. (Shanghai, China).

### 4.2. Animals and Experimental Protocols

This study received approval from the Animal Ethics Committee of Fujian University of Traditional Chinese Medicine (Ethical code: 2022017), and all experiments were conducted in accordance with the “Guide for the Care and Use of Laboratory Animals” (National Institutes of Health) [75]. Male C57BL/6 mice (22~25 g, 8 weeks old) were procured from SLAC Laboratory Animal Technology Co. Ltd. (Shanghai, China). The mice were randomly divided into three groups: Control, Ang II, and Ang II + gastrodin, with six mice in each group. Gastrodin was dissolved in sterilized ddH_2_O and intragastrically administered to mice daily at a final concentration of 5 mg/kg. Infusions with Ang II (500 ng/kg/min) and gastrodin treatment (5 mg/kg/day) were administered for 4 weeks according to the protocol from our previous study [32,33]. 

### 4.3. Blood Pressure Measurement and Echocardiography

Blood pressure in the mice was measured by an individual who was blinded to the experimental groups, using the tail-cuff plethysmograph method as outlined in our previous study [32]. Cardiac parameters of mice anesthetized with 1.5% isoflurane were assessed by the Vevo2100 High-Resolution Imaging System (Visual Sonics, Toronto, ON, Canada). The ultrasound probe (30 MHz) was placed at a 45-degree angle from the midline of the sternum to visualize the long axis of the left ventricle [44].

### 4.4. Determination of Heart Weight/Tibia Length Ratio and Hematoxylin and Eosin (H&E) Staining

At the end of the animal experiment, the mice were euthanized by cervical dislocation, and their hearts were removed. The heart weight (HW) and tibia length (TL) were measured to calculate the HW/TL ratio. The hearts were then longitudinally sectioned into three equal parts. The left part of heart was used for RNA sequencing, the central part of heart was embedded in paraffin for H&E staining, immunohistochemical (IHC) analysis, and TUNEL assays, while the right part of heart was used for Western blot. Six mice from each group were used for RNA sequencing, H&E, IHC, and TUNEL assays. Three mice from each group were randomly selected, and their cardiac tissues were used for Western blot analysis. 

For the H&E staining experiment, the cardiac tissues were rinsed with normal saline, fixed in 4% paraformaldehyde, embedded in paraffin, and sliced into 4-μm thick sections. These sections were stained with hematoxylin for 45 s and eosin for 5 s. Finally, the stained sections were imaged using a Leica DM6000B microscope (Leica Microsystems, Wetzlar, Germany) at 400× magnification. Then, the stained sections from the same region of the heart from different mice were used for comparison.

### 4.5. Immunohistochemical (IHC) Analysis

The cardiac sections from the same region of the heart from different mice were dewaxed for antigenic retrieval and then blocked with 3% hydrogen peroxide at room temperature for 10 min. The sections were incubated with antibodies against PRDX2 (dilution, 1:500), p53 (dilution, 1:500) and anti-mouse IgG (dilution: 1:5000). After incubation, the sections were treated with diaminobenzidine and counterstained with diluted hematoxylin. The images were captured using an intelligent scanning system (Leica DM6000B) at 400× magnification. Finally, five random fields per sample of the positively stained area (immunostained area based on RGB value) in each field were analyzed using Image J (version 2022) software [76]. 

### 4.6. TUNEL Staining

Cell apoptosis in the cardiac tissues of mice was assessed using TUNEL staining following the manufacturer’s instructions. Briefly, the cardiac sections were incubated in proteinase K solution at room temperature for 15 min. Subsequently, they were treated with the TUNEL reaction mixture (50 μL), converter peroxidase (50 μL), and the diaminobenzidine substrate (100 μL), respectively. After counterstaining with hematoxylin, the sections were imaged at 400× magnification. Finally, five fields per sample of the positively stained cells were analyzed using Image J (version 2022) software.

### 4.7. RNA Sequencing (RNA-Seq)

Cardiac tissues of mice were preserved in an RNA-protective solution (Takara, Beijing, China, cat. no. 9750) at room temperature for 2 h and then transferred to −80 °C for long-term storage. Total RNA was extracted using Trizol reagent (Thermo Fisher Scientific, cat. no. 15596026). RNA concentration and quality were assessed using a Qubit 3.0 and Agilent 2100 Bioanalyzer. Only RNA samples with RNA Integrity Numbers (RINs) of 7 or higher were used for further experiments.

RNA-seq was performed as previously described by CapitalBio Technology (Beijing, China) [77,78]. Differentially expressed transcripts (DETs) were identified using DESeq (v1.28.0). Gene Ontology (GO) and the Kyoto Encyclopedia of Genes and Genomes (KEGG) analyses were conducted to further explore the signaling pathways of the DETs.

### 4.8. Cell Culture and Treatments

The cardiomyocyte cell line H9c2 was obtained and cultured as described previously [44]. A 100 mM stock solution of gastrodin was prepared with sterilized ddH_2_O just before use in the cell experiment. H9c2 cells were starved for 12 h, then treated with different concentrations of gastrodin (0, 10, 20, 40, 80, 120, 160 μM) for 24 h in 96-well plates. Then, the cell viability was detected using CCK8 assays, following the manufacturer’s instructions (Abbkine Scientific Co., Ltd., Wuhan, China). Three non-toxic doses of gastrodin (20, 40, and 80 μM) were selected for subsequent in vitro experiments (Figure S2). H9c2 cells were starved for 12 h and then divided into three groups: Control (ddH2O), Ang II (0.1 μM), and Ang II (0.1 μM) + gastrodin (20, 40, or 80 μM). The cells were treated with Ang II and gastrodin, prepared in high-glucose DMEM medium containing 2.5 % FBS for 24 h.

### 4.9. Hoechst Staining

H9c2 cells treated with Ang II (0.1 μM) and/or gastrodin (20, 40 or 80 μM) were fixed with a 4% paraformaldehyde solution and stained with Hoechst for 10 min. The cells were then imaged using a laser confocal microscope (PerkinElmer, Santa Clara, CA, USA) at a magnification of 200×.

### 4.10. Apoptosis Assay by Flow Cytometry

H9c2 cells treated with Ang II (0.1 μM) and/or gastrodin (20, 40 or 80 μM) were collected and washed with cold PBS. The cells were then suspended in 1× Binding Buffer and stained with FITC Annexin V and propidium iodide (PI) for 15 min. The stained cells were subsequently analyzed using a Flow cytometer (BD, Franklin Lakes, NJ, USA). 

### 4.11. Western-Blot Analysis

Total protein was extracted from the cardiac tissues of the same regions from different mice or H9c2 cells using a cell lysis buffer containing protease and phosphatase inhibitors, following the manufacturer’s instructions. The BCA kit was used to determine the concentration of protein. A total of 50 μg protein was separated on 10% SDS-PAGE gels and transferred to a Polyvinylidene fluoride (PVDF) membrane. The membranes were blocked with a sealing solution for 2 h and then incubated with the primary antibody (PRDX2, p53, Bax, Bcl-2, cleaved caspase-3, cleaved caspase-9, or β-actin; all at a dilution: 1:1000) over night. Subsequently, the membranes were incubated with secondary antibody (rabbit or mouse; dilution: 1:5000). The band intensity was detected using the ChemiDoc XRS + imaging system (Bio-Rad Laboratories, Inc., Hercules, CA, USA) and quantified with Image J (version 2022) software.

### 4.12. Statistical Analysis

Statistical analysis was conducted using SPSS 22.0 software (SPSS/PC+, Chicago, IL, USA). All data were expressed as the mean ± standard deviation (SD). All statistical analyses were conducted by experimenters who were blinded to the treatment conditions. The assumptions of normality and homogeneity of variance were first assessed. For data following a normal distribution, one-way analysis of variance (ANOVA) was applied. When ANOVA indicated significant differences among groups, data with equal variances were analyzed using the LSD test, and data with unequal variances were analyzed by Games–Howell analysis. For data not conforming to a normal distribution (data in Figure 2f), the Mann–Whitney U test or the Kruskal–Wallis test (depending on the number of groups) was applied, followed by Dunn’s test for pairwise comparisons. A *p*-value of less than 0.05 was considered statistically significant.

## 5. Conclusions

Our study demonstrated that gastrodin effectively alleviated Ang II-induced blood pressure elevation, cardiac dysfunction, and pathological changes, as well as reduced hypertension-induced myocardial apoptosis in hypertensive mice induced by Ang II in vivo and H9c2 cells stimulated by Ang II in vitro. Furthermore, we found that inhibition of PRDX2/p53 pathway may be one of the regulatory mechanisms by which gastrodin reduces Ang II-induced hypertension and exerts cardioprotective effects.

## Figures and Tables

**Table 1:** Effect of gastrodin on endpoint systolic blood pressure (SBP) in Ang II-induced hypertensive mice.

Group	Mean ± SD	Median	IQRs ^1^ (25%)	IQRs (75%)
Control	104.55 ± 2.69	104.83	103.79	99.91
Ang II	178.47 ± 10.96	174.54	172.60	169.09
Ang II + gastrodin	126.34 ± 6.31	125.44	123.28	117.89

^1^ IQRs: Interquartile ranges.

**Table 2:** Effect of gastrodin on endpoint mean arterial pressure (DBP) in Ang II-induced hypertensive mice.

Group	Mean ± SD	Median	IQRs ^1^ (25%)	IQRs (75%)
Control	71.93 ± 2.68	72.37	71.27	67.18
Ang II	154.04 ± 7.60	150.57	149.24	147.93
Ang II + gastrodin	105.72 ± 7.34	104.85	99.47	98.00

^1^ IQRs: Interquartile ranges.

**Table 3:** Effect of gastrodin on endpoint systolic blood pressure (MAP) in Ang II-induced hypertensive mice.

Group	Mean ± SD	Median	IQRs ^1^ (25%)	IQRs (75%)
Control	86.20 ± 1.74	86.68	85.46	83.27
Ang II	164.21 ± 7.87	161.24	158.85	158.13
Ang II + gastrodin	112.76 ± 7.59	111.90	106.89	104.33

^1^ IQRs: Interquartile ranges.

## Data Availability

All data can be found within the article and its supplementary information. RNA-seq date are available through the GEO database, accession number GSE193504.

## Supplementary Materials

The following supporting information can be downloaded at: https://www.mdpi.com/article/10.3390/ph17091200/s1, Figure S1: The chemical structure of gastrodin. The chemical structure of gastrodin was downloaded from PubChem (https://pubchem.ncbi.nlm.nih.gov/compound/115067). Figure S2: Relative cell viability of H9c2 cells following the treatment of gastrodin.

## Abbreviations

Ang II: angiotensin II; BCA, bicinchoninic acid; BPs, biological processes; CC, cellular composition; DBP, diastolic blood pressure; IQRs, Interquartile ranges; DETs, differentially expressed transcripts; DMEM, Dulbecco’s modified Eagle’s medium; FBS, fetal bovine serum; GO, Gene Ontology; H&E, hematoxylin and eosin; HW, heart weight; IHC, immunohistochemistry; KEGG, Kyoto Encyclopedia of Genes and Genomes; LVEF, left ventricle ejection fraction; LVFS, left ventricle fractional shortening; LV Mass, corrected left ventricular mass; LV Vol. d, left ventricle volume diastole; LV Vol. s, left ventricle volume systole; MAP, mean arterial pressure; MF, molecular function; PBS, phosphate-buffered saline; PI, propidium iodide; PVDF, Polyvinylidene fluoride; RAAS, renin–angiotensin–aldosterone system; RNA-seq, RNA sequencing; SBP, systolic blood pressure; TL, tibia length; TUNEL, terminal deoxynucleotidyl transferase-mediated nick end labeling; mPTP, mitochondrial permeability transition pore; LPS/D-GalN, Lipopolysaccharide/D-galactosamine.

## References

[1] Jarraya F. (2017). Treatment of Hypertension: Which Goal for Which Patient?. Adv. Exp. Med. Biol..

[2] Riaz S., Zeidan A., Mraiche F. (2017). Myocardial proteases and cardiac remodeling. J. Cell Physiol..

[3] Li X., Pu Z., Xu G., Yang Y., Cui Y., Zhou X., Wang C., Zhong Z., Zhou S., Yin J. (2024). Hypoxia-Induced Myocardial Hypertrophy Companies with Apoptosis Enhancement and p38-MAPK Pathway Activation. High Alt. Med. Biol..

[4] Cheng Y., Yan M., He S., Xie Y., Wei L., Xuan B., Shang Z., Wu M., Zheng H., Chen Y. (2024). Baicalin alleviates angiotensin II-induced cardiomyocyte apoptosis and autophagy and modulates the AMPK/mTOR pathway. J. Cell. Mol. Med..

[5] Wang X., Simpson E.R., Brown K.A. (2015). p53: Protection against Tumor Growth beyond Effects on Cell Cycle and Apoptosis. Cancer Res..

[6] Sano M., Minamino T., Toko H., Miyauchi H., Orimo M., Qin Y., Akazawa H., Tateno K., Kayama Y., Harada M. (2007). P53-induced Inhibition of Hif-1 Causes Cardiac Dysfunction during Pressure Overload. Nature.

[7] Yano T., Abe K., Tanno M., Miki T., Kuno A., Miura T., Steenbergen C. (2018). Does p53 Inhibition Suppress Myocardial Ischemia-Reperfusion Injury?. J. Cardiovasc. Pharm. Ther..

[8] Hsieh S.Y., Hsu C.Y., He J.R., Liu C.L., Lo S.J., Chen Y.C., Huang H.Y. (2009). Identifying apoptosis-evasion proteins/pathways in human hepatoma cells via induction of cellular hormesis by UV irradiation. J. Proteome Res..

[9] Qian L., Yan S., Li Y., Wu L., Zheng Y., Wang Y., Fang Z. (2020). The effects of gastrodin injection on hypertension: A systematic review and meta-analysis. Medicine.

[10] Alshalalfeh M., Sun N., Moraes A.H., Utani A.P.A., Xu Y. (2023). Conformational Distributions of Phenyl β-D-Glucopyranoside and Gastrodin in Solution by Vibrational Optical Activity and Theoretical Calculations. Molecules.

[11] Huang B., Lin Z., Chen Z., Chen J., Shi B., Jia J., Li Y., Pan Y., Liang Y., Cai Z. (2023). Strain differences in the drug transport capacity of intestinal glucose transporters in Sprague-Dawley versus Wistar rats, C57BL/6J versus Kunming mice. Int. J. Pharm..

[12] Zhao Y., Gong X.J., Zhou X., Kang Z.J. (2014). Relative bioavailability of gastrodin and parishin from extract and powder of Gastrodiae rhizoma in rat. J. Pharm. Biomed. Anal..

[13] Lv Y., Cao H., Chu L., Peng H., Shen X., Yang H. (2021). Effects of Gastrodin on BV2 cells under oxygen-glucose deprivation and its mechanism. Gene.

[14] Hu Y., Li C., Shen W. (2014). Gastrodin alleviates memory deficits and reduces neuropathology in a mouse model of Alzheimer’s disease. Neuropathology.

[15] Han X., Shi H., Liu K., Zhong L., Wang F., You Q. (2019). Protective effect of gastrodin on myocardial ischemia-reperfusion injury and the expression of Bax and Bcl-2. Exp. Ther. Med..

[16] Fu S., Chen L., Wu Y., Tang Y., Tang L., Zhong Y., Wang S., Liu H., Wang X., Chen A. (2018). Gastrodin pretreatment alleviates myocardial ischemia/reperfusion injury through promoting autophagic flux. Biochem. Biophys. Res. Commun..

[17] Zhang M., Tan Y., Song Y., Zhu M., Zhang B., Chen C., Wang S., Liu H., Wang X., Chen A. (2023). GLUT4 mediates the protective function of gastrodin against pressure overload-induced cardiac hypertrophy. Biomed. Pharmacother..

[18] Zheng C., Lo C.Y., Meng Z., Li Z., Zhong M., Zhang P., Lu J., Yang Z., Yan F., Zhang Y. (2017). Gastrodin Inhibits Store-Operated Ca^2+^ Entry and Alleviates Cardiac Hypertrophy. Front. Pharmacol..

[19] Chen J., Huang Y., Hu X., Bian X., Nian S. (2021). Gastrodin prevents homocysteine-induced human umbilical vein endothelial cells injury via PI3K/Akt/eNOS and Nrf2/ARE pathway. J. Cell. Mol. Med..

[20] Chen J., Gu Y.T., Xie J.J., Wu C.C., Xuan J., Guo W.J., Yan Y.Z., Chen L., Wu Y.S., Zhang X.L. (2018). Gastrodin reduces IL-1β-induced apoptosis, inflammation, and matrix catabolism in osteoarthritis chondrocytes and attenuates rat cartilage degeneration in vivo. Biomed. Pharmacother..

[21] Zhang T.H., Huang C.M., Gao X., Wang J.W., Hao L.L., Ji Q. (2018). Gastrodin inhibits high glucose-induced human retinal endothelial cell apoptosis by regulating the SIRT1/TLR4/NF-κBp65 signaling pathway. Mol. Med. Rep..

[22] Cheng Q.-Q., Wan Y.-W., Yang W.-M., Tian M.-H., Wang Y.-C., He H.-Y., Zhang W.D., Liu X. (2020). Gastrodin protects H9c2 cardiomyocytes against oxidative injury by ameliorating imbalanced mitochondrial dynamics and mitochondrial dysfunction. Acta Pharmacol. Sin..

[23] Wang W., Wei J., Zhang H., Zheng X., Zhou H., Luo Y., Yang J., Deng Q., Huang S., Fu Z. (2021). PRDX2 promotes the proliferation of colorectal cancer cells by increasing the ubiquitinated degradation of p53. Cell Death Dis..

[24] Wu F., Tian F., Zeng W., Liu X., Fan J., Lin Y., Zhang Y. (2017). Role of peroxiredoxin2 downregulation in recurrent miscarriage through regulation of trophoblast proliferation and apoptosis. Cell Death Dis..

[25] Shao J.H., Fu Q.W., Li L.X., Zhou R., Liu N., Peng J.H., Chen Y. (2020). Prx II reduces oxidative stress and cell senescence in chondrocytes by activating the p16-CDK4/6-pRb-E2F signaling pathway. Eur. Rev. Med. Pharmacol. Sci..

[26] Huang Z., Chen Z., Wang X., Ding X., Cai Z., Li W., Cai Z., Lan Y., Chen G., Fang W. (2022). Association of Cardiovascular Health Score Trajectory With Incident Myocardial Infarction in Hypertensive Patients. Hypertension.

[27] Xiao G., Tang R., Yang N., Chen Y. (2023). Review on pharmacological effects of gastrodin. Arch. Pharm. Res..

[28] Zhang H., Wang L., Lu B., Qi W., Jiao F., Zhang H., Yuan D. (2019). Metabolite profiling and quantification of phytochemicals of Tianma-Gouteng granule in human and rat urine using ultra high performance liquid chromatography coupled with tandem mass spectrometry. J. Sep. Sci..

[29] Zhang H., Duan S., Wang L., Liu J., Qi W., Yuan D. (2019). Identification of the absorbed components and their metabolites of Tianma-Gouteng granule in rat plasma and bile using ultra-high-performance liquid chromatography combined with quadrupole time-of-flight mass spectrometry. Biomed. Chromatogr..

[30] Li Y., Li F. (2022). Mechanism and Prospect of Gastrodin in Osteoporosis, Bone Regeneration, and Osseointegration. Pharmaceuticals.

[31] Zheng Y.Y., Dong Z., Lu X.Q., Xia Y.P., Zhu S.B. (2015). Analysis on 315 cases of clinical adverse drug reaction/event induced by gastrodin. Zhongguo Zhong Yao Za Zhi.

[32] Guo Z., Yang X., Wu M., Shen A., Li J., Zhang X., Cheng Y., Xie Q., Peng J. (2023). Gastrodin attenuates angiotensin II-induced vascular contraction and MLCK/p-MLC(2) pathway activation. Pharm. Biol..

[33] Wen Y., Zhang X., Wei L., Wu M., Cheng Y., Zheng H., Shen A., Fu C., Ali F., Long L. (2023). Gastrodin attenuates renal injury and collagen deposition via suppression of the TGF-β1/Smad2/3 signaling pathway based on network pharmacology analysis. Front. Pharmacol..

[34] Shen A., Wu M., Ali F., Guo Z., Fang Y., Zhou Y., Zhang S., Zhang W., Wen Y., Yu M. (2023). Based on network pharmacology, gastrodin attenuates hypertension-induced vascular smooth muscle cell proliferation and PI3K/AKT pathway activation. Sci. Rep..

[35] Liu W., Su B.L., Wang Z.S., Zhang X., Gao Y.S., Song S.W. (2012). Gastrodin improved baroreflex sensitivity and increased gamma-amino butyric acid content in brains without decreasing blood pressure in spontaneously hypertensive rats. CNS Neurosci. Ther..

[36] Zhu M., Deng W., Di S., Qin M., Liu D., Yi B. (2018). Gastrodin Protects Cardiomyocytes from Anoxia/Reoxygenation Injury by 14-3-3*η*. Oxid. Med. Cell. Longev..

[37] Shu C., Chen C., Zhang D.P., Guo H., Zhou H., Zong J., Bian Z., Dong X., Dai J., Zhang Y. (2012). Gastrodin protects against cardiac hypertrophy and fibrosis. Mol. Cell. Biochem..

[38] Vermot A., Petit-Härtlein I., Smith S.M.E., Fieschi F. (2021). NADPH Oxidases (NOX): An Overview from Discovery, Molecular Mechanisms to Physiology and Pathology. Antioxidants.

[39] Liu S.J., Liu X.Y., Li J.H., Guo J., Li F., Gui Y., Li X.H., Yang L., Wu C.Y., Yuan Y. (2018). Gastrodin attenuates microglia activation through renin-angiotensin system and Sirtuin3 pathway. Neurochem. Int..

[40] Jalmi S.K., Sinha A.K. (2015). ROS mediated MAPK signaling in abiotic and biotic stress- striking similarities and differences. Front. Plant Sci..

[41] Xu J.M., Tan R., Hu D.X. (2003). Effect of ischemic preconditioning on myocardial bcl-2, bax, p53 gene expression during ischemia/reperfusion period in rabbits. Hunan Yi Ke Da Xue Xue Bao.

[42] Kai H., Kudo H., Takayama N., Yasuoka S., Kajimoto H., Imaizumi T. (2009). Large blood pressure variability and hypertensive cardiac remodeling—Role of cardiac inflammation. Circ. J..

[43] Huang X.R., Chung A.C., Yang F., Yue W., Deng C., Lau C.P., Tse H.F., Lan H.Y. (2010). Smad3 mediates cardiac inflammation and fibrosis in angiotensin II-induced hypertensive cardiac remodeling. Hypertension.

[44] Cheng Y., Shen A., Wu X., Shen Z., Chen X., Li J., Liu L., Lin X., Wu M., Chen Y. (2021). Qingda granule attenuates angiotensin II-induced cardiac hypertrophy and apoptosis and modulates the PI3K/AKT pathway. Biomed. Pharmacother..

[45] González A., Fortuño M.A., Querejeta R., Ravassa S., López B., López N., Díez J. (2003). Cardiomyocyte apoptosis in hypertensive cardiomyopathy. Cardiovasc. Res..

[46] Shao F., Zhou L., Zhang Y., Chen H., Zhang Y., Guan Z. (2021). Gastrodin alleviates inflammatory injury of cardiomyocytes in septic shock mice via inhibiting NLRP3 expression. In Vitro Cell. Dev. Biol. Anim..

[47] Chunhacha P., Pinkaew D., Sinthujaroen P., Bowles D.E., Fujise K. (2021). Fortilin inhibits p53, halts cardiomyocyte apoptosis, and protects the heart against heart failure. Cell Death Discov..

[48] Wang Z., Yang K., Zheng Q., Zhang C., Tang H., Babicheva A., Jiang Q., Li M., Chen Y., Carr S. (2019). Divergent changes of p53 in pulmonary arterial endothelial and smooth muscle cells involved in the development of pulmonary hypertension. Am. J. Physiol.-Lung Cell. Mol. Physiol..

[49] Zhang Z.Y., Li Y., Li R., Zhang A.A., Shang B., Yu J., Xie X. (2016). Tetrahydrobiopterin Protects against Radiation-induced Growth Inhibition in H9c2 Cardiomyocytes. Chin. Med. J..

[50] Shan R., Zhang Y., Shi Y., Wang X., Wang X., Ma G., Li Q. (2024). Activation of Cannabinoid Type 2 Receptor in Microglia Reduces Neuroinflammation through Inhibiting Aerobic Glycolysis to Relieve Hypertension. Biomolecules.

[51] Wu F., Zuo H.J., Ren X.Q., Wang P.X., Li F., Li J.J. (2023). Gastrodin Regulates the Notch-1 Signal Pathway via Renin-Angiotensin System in Activated Microglia. NeuroMol. Med..

[52] Wang X., Lin L., Lan B., Wang Y., Du L., Chen X., Li Q., Liu K., Hu M., Xue Y. (2020). IGF2R-initiated proton rechanneling dictates an anti-inflammatory property in macrophages. Sci. Adv..

[53] Lu J., Ma X., Gao W.C., Zhang X., Fu Y., Liu Q., Tian L., Qin X., Yang W., Zheng H. (2021). Gastrodin Exerts Cardioprotective Action via Inhibition of Insulin-Like Growth Factor Type 2/Insulin-Like Growth Factor Type 2 Receptor Expression in Cardiac Hypertrophy. ACS Omega.

[54] Wang X.L., Xing G.H., Hong B., Li X.M., Zou Y., Zhang X.J., Dong M.X. (2014). Gastrodin prevents motor deficits and oxidative stress in the MPTP mouse model of Parkinson’s disease: Involvement of ERK1/2-Nrf2 signaling pathway. Life Sci..

[55] Lin J., Shi Y., Miao J., Wu Y., Lin H., Wu J., Zeng W., Qi F., Liu C., Wang X. (2019). Gastrodin Alleviates Oxidative Stress-Induced Apoptosis and Cellular Dysfunction in Human Umbilical Vein Endothelial Cells via the Nuclear Factor-Erythroid 2-Related Factor 2/Heme Oxygenase-1 Pathway and Accelerates Wound Healing In Vivo. Front. Pharmacol..

[56] Tang L.J., Zhou Y.J., Xiong X.M., Li N.S., Zhang J.J., Luo X.J., Peng J. (2021). Ubiquitin-specific protease 7 promotes ferroptosis via activation of the p53/TfR1 pathway in the rat hearts after ischemia/reperfusion. Free Radic. Biol. Med..

[57] Xing P., Li X., Bai Y., Jiao Z. (2023). Cypermethrin and/or sulfamethoxazole exposure effect on apoptosis and endoplasmic reticulum of grass carp cardiomyocyte. Ecotoxicol. Environ. Saf..

[58] Shen Z., Shen A., Chen X., Wu X., Chu J., Cheng Y., Peng M., Chen Y., Weygant N., Wu M. (2020). Huoxin pill attenuates myocardial infarction-induced apoptosis and fibrosis via suppression of p53 and TGF-β1/Smad2/3 pathways. Biomed. Pharmacother..

[59] Cao Y., Chen Z., Jia J., Chen A., Gao Y., Qian J., Ge J. (2022). Rosuvastatin Alleviates Coronary Microembolization-Induced Cardiac Injury by Suppressing Nox2-Induced ROS Overproduction and Myocardial Apoptosis. Cardiovasc. Toxicol..

[60] Jiang G., Wu H., Hu Y., Li J., Li Q. (2014). Gastrodin inhibits glutamate-induced apoptosis of PC12 cells via inhibition of CaMKII/ASK-1/p38 MAPK/p53 signaling cascade. Cell. Mol. Neurobiol..

[61] Lv H., Liu Y., Zhang B., Zheng Y., Ji H., Li S. (2020). The improvement effect of gastrodin on LPS/GalN-induced fulminant hepatitis via inhibiting inflammation and apoptosis and restoring autophagy. Int. Immunopharmacol..

[62] Singh A., Tandon S., Tandon C. (2021). An update on vascular calcification and potential therapeutics. Mol. Biol. Rep..

[63] Lv C., Huang Y., Wang Q., Wang C., Hu H., Zhang H., Lu D., Jiang H., Shen R., Zhang W. (2023). Ainsliadimer A induces ROS-mediated apoptosis in colorectal cancer cells via directly targeting peroxiredoxin 1 and 2. Cell Chem. Biol..

[64] Jin X., Chen C., Li D., Su Q., Hang Y., Zhang P., Hu W. (2017). PRDX2 in Myocyte Hypertrophy and Survival is Mediated by TLR4 in Acute Infarcted Myocardium. Sci. Rep..

[65] Li J., Wang C., Wang W., Liu L., Zhang Q., Zhang J., Wang B., Wang S., Li H., Gao C. (2021). PRDX2 Protects Against Atherosclerosis by Regulating the Phenotype and Function of the Vascular Smooth Muscle Cell. Front. Cardiovasc. Med..

[66] Li Y., Ji Y., Li F. (2023). A review: Mechanism and prospect of gastrodin in prevention and treatment of T2DM and COVID-19. Heliyon.

[67] Zhou Y., Li M., Lv T., Huang M., Cheng B., Zhang Y., Zhu J. (2020). Gastrodin Inhibits Virus Infection by Promoting the Production of Type I Interferon. Front. Pharmacol..

[68] Liu Z., Wang S., Zhang J., Wang Y., Wang Y., Zhang L., Zhang L., Li L., Dong J., Wang B. (2019). Gastrodin, a traditional Chinese medicine monomer compound, can be used as adjuvant to enhance the immunogenicity of melanoma vaccines. Int. Immunopharmacol..

[69] Shu G., Yang T., Wang C., Su H., Xiang M. (2013). Gastrodin stimulates anticancer immune response and represses transplanted H22 hepatic ascitic tumor cell growth: Involvement of NF-κB signaling activation in CD4+ T cells. Toxicol. Appl. Pharmacol..

[70] Li L., Li Q., Gui L., Deng Y., Wang L., Jiao J., Hu Y., Lan X., Hou J., Li Y. (2023). Sequential gastrodin release PU/n-HA composite scaffolds reprogram macrophages for improved osteogenesis and angiogenesis. Bioact. Mater..

[71] Wei L., Wang L., Yu J., Fordjour A.P., Zhao Y.Q. (2015). Gastrodin Reduces Blood Pressure by Intervening with RAAS and PPARγ in SHRs. Evid.-Based Complement. Altern. Med..

[72] Yang H., Li Q., Li L., Chen S., Zhao Y., Hu Y., Wang L., Lan X., Zhong L., Lu D. (2022). Gastrodin modified polyurethane conduit promotes nerve repair via optimizing Schwann cells function. Bioact. Mater..

[73] Yongguang L., Xiaowei W., Huichao Y., Yanxiang Z. (2022). Gastrodin promotes the regeneration of peripheral nerves by regulating miR-497/BDNF axis. BMC Complement. Med..

[74] Liu C.M., Tian Z.K., Zhang Y.J., Ming Q.L., Ma J.Q., Ji L.P. (2020). Effects of Gastrodin against Lead-Induced Brain Injury in Mice Associated with the Wnt/Nrf2 Pathway. Nutrients.

[75] Ye T., Meng X., Wang R., Zhang C., He S., Sun G., Sun X. (2018). Gastrodin Alleviates Cognitive Dysfunction and Depressive-Like Behaviors by Inhibiting ER Stress and NLRP3 Inflammasome Activation in db/db Mice. Int. J. Mol. Sci..

[76] Shu J., Qiu G., Mohammad I. (2013). A Semi-Automatic Image Analysis Tool for Biomarker Detection in Immunohistochemistry Analysis. Proceedings of the 2013 Seventh International Conference on Image and Graphics.

[77] Long L., Zhang X., Wen Y., Li J., Wei L., Cheng Y., Liu H., Chu J., Fang Y., Xie Q. (2021). Qingda Granule Attenuates Angiotensin II-Induced Renal Apoptosis and Activation of the p53 Pathway. Front. Pharmacol..

[78] Shen Y., White E. (2001). p53-dependent apoptosis pathways. Adv. Cancer Res..

