# Supplementary material for: Gastrodin Alleviates Angiotensin II-Induced Hypertension and Myocardial Apoptosis via Inhibition of the PRDX2/p53 Pathway In Vivo and In Vitro
Source: Pharmaceuticals (Basel). 2024 Sep 12;17(9):1200. doi: 10.3390/ph17091200 (PMC11434704; doi:10.3390/ph17091200)
Supplement: Supplementary file 1 [file pharmaceuticals-17-01200-s001.zip › pharmaceuticals-3131985-supplementary.pdf]

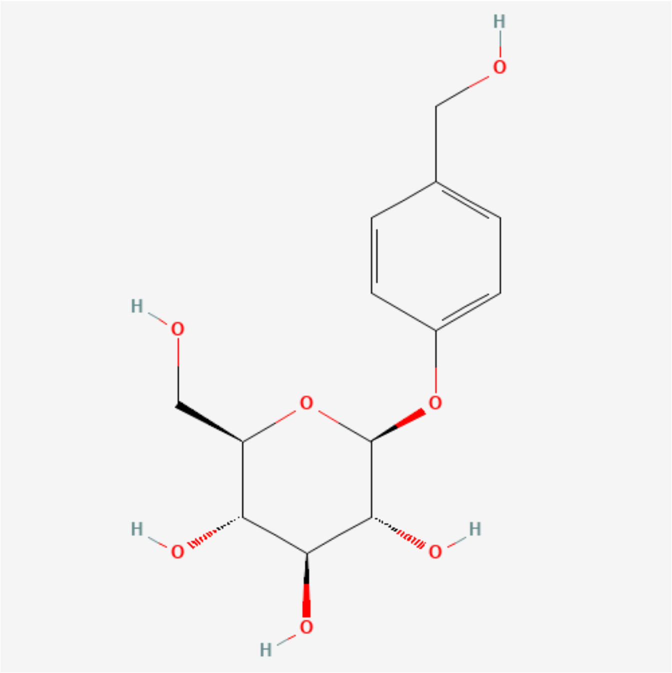

Figure S1: The chemical structure of gastrodin. The chemical structure of gastrodin was downloaded from PubChem (https://pubchem.ncbi.nlm.nih.gov/compound/115067).

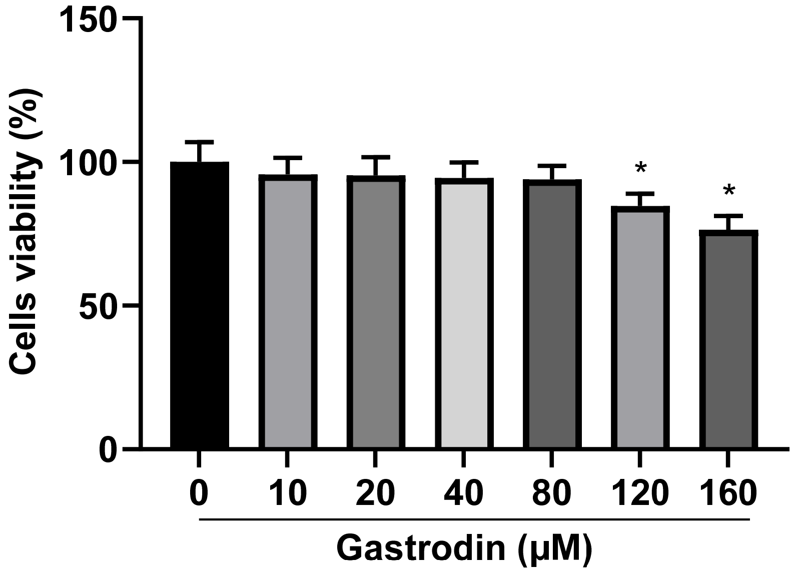

Figure S2: Relative cell viability of H9c2 cells following the treatment of gastrodin.
